# Dietary patterns and primary liver cancer in Chinese adults: a case-control study

**DOI:** 10.18632/oncotarget.23910

**Published:** 2018-01-04

**Authors:** Qiu-Ye Lan, Gong-Cheng Liao, Rui-Fen Zhou, Pei-Yan Chen, Xiao-Yan Wang, Min-Shan Chen, Yu-Ming Chen, Hui-Lian Zhu

**Affiliations:** ^1^ School of Public Health, Sun Yat-Sen University, Guangzhou 510080, People's Republic of China; ^2^ Department of Hepatobiliary Oncology, Sun Yat-Sen University Cancer Center, Guangzhou 510060, People's Republic of China

**Keywords:** primary liver cancer, factor analysis, dietary pattern

## Abstract

**Introduction:**

Healthy dietary patterns may prevent many chronic diseases, and is emphasized by 2015 US dietary guideline, but it remains unclear which dietary patterns may be benefit to prevention of primary liver cancer (PLC).

**Materials and Methods:**

We recruited 782 PLC cases and 1:1 age- and sex-matched controls in Guangzhou, China. Habitual dietary intake was assessed by face-to-face interview using a 79-item food frequency questionnaire, and used to explore dietary patterns by factor analysis.

**Results:**

Three dietary patterns were identified: 1) an urban prudent dietary pattern (UPDP) characterized by high in dairy products, eggs, mushrooms, nuts and soy foods, but low in refined grains; 2) a traditional Cantonese dietary pattern (TCDP) consisting of a high intake of fruit and vegetables, fish, Cantonese soup, and Chinese herb tea; and 3) a high meat and preserved food pattern (MPFP). Multivariable analyses showed favorable associations for the first two dietary patterns, but unfavorable association for the last one (all *p*-trend < 0.01). Odds ratios (95% CI) of PLC for the highest (vs. lowest) quartile of pattern scores of the three patterns were 0.25 (0.18–0.35), 0.61 (0.46–0.82), and 1.98 (1.46–2.69), respectively.

**Conclusions:**

Our findings suggest that the UPDP and TCDP were associated with lower, whereas the MPFP with higher, risk of PLC.

## INTRODUCTION

In 2013, primary liver cancer (PLC) ranked sixth in cancer incidence and third in mortality worldwide and the rates of death from PLC of both sexes increased from 2003 to 2012 [1, 2]. PLC is also one of the most commonly diagnosed cancers in China. The main risk factor for hepatocellular carcinoma is HBV or HCV infection [3], but other prevention strategies relating to dietary or lifestyle factors [4–6] have also been proven in previous studies to be crucial.

Previous studies have showed that many foods and nutrients are associated with the risk of PLC. For example, carbohydrates [7], foods from animal sources with rich saturated fat (e.g., meat [8, 9], and dairy products [10]), alcohol [11] and soft drinks [12] tend to increase the PLC risk, while vitamins [13], dietary fiber [7], a great deal of foods from plant sources [14], coffee [15, 16] and fish [17, 18] tend to decrease the risk. They provide comprehensive knowledge of the effect of a single/type food or nutrient on the diseases. However, people usually consumes complex foods and nutrients in a diet. It is hard to identify the independent effect of single food/nutrient, and to perform the dietary intervention with a single food. Dietary pattern analysis provides an integral view of an individual’s diet, and studying the interactions between nutrients and foods was thus proposed as an alternative approach [19]. In 2015, the US Dietary Guideline mainly focused on healthy dietary patterns, but not individual foods [20]. A large number of studies have identified various dietary patterns that may be benefit to the cancer prevention or increase some cancer risks. Flood *et al*’s study based on the National Institutes of Health-AARP Diet and Health study reported a fruit and vegetable dietary pattern was associated with a decreased risk of colorectal cancer [21]. Similar favorable results about plant-diet on colorectal cancer [22] or breast cancer [23] were also found. But a study from Japan demonstrated a Westernized diet might increase the risk of breast cancer [24]. However, few studies have examined the relationship between data-driven dietary patterns and the risk of PLC. Considering dietary patterns might be disease-specific, more studies are needed for PLC. In addition, dietary patterns tend to be diverse across different geographic areas and socioeconomic statuses [25]. Chinese populaitons have diverse diets among different regions, and are quite different from their Western counterparts. In view of great population of newly diagnosed liver cancer cases (466.1 thousand per year)in China [26], it is urgent to identify the potential healthy dietary patterns for the prevention of PLC in Chinese population. Thus, the aim of our study was to identify the dietary patterns based on factor analysis using a food frequency questionnaire and to explore their potential relation to PLC risk within a Chinese population residing in Guangdong province.

## RESULTS

The basic characteristics of the cases and controls are shown in Table 1. There was a total of 782 case-control pairs, 102 female pairs and 680 male pairs. There was no significant difference in the median age between cases and controls. Compared with the control subjects, PLC patients were more likely to have lower values of BMI, WHR, physical activity, education level, and energy intake, but higher household income and higher proportions of married people, smokers, alcohol users and individuals with diabetes (all *P* < 0.05). No significant differences were found between the case and control subjects in tea consumption, multivitamin use or hypertension status. 86.7% of the PLC patients were HBV infectious. Among the case subjects with tissue evaluation, 637 (96.4%) were hepatocellular carcinoma (HCC), 19 (2.9%) were cholangiocarcinoma, and 5 (0.8%) were combined hepatocellular-cholangiocarcinoma.

**Table 1:** Comparison of general characteristics between primary liver cancer cases and controls

Characteristics	PLC (*n =* 782)	Controls (*n =* 782)	*P*
Age (years)^1^	52.71 (11.27)	53.02 (10.20)	0.568^*^
Gender (%)			
Male	680 (87.0)	680 (87.0)	1^#^
Female	102 (13.0)	102 (13.0)	
BMI (kg/m^2^)^1^	22.81 (3.31)	23.25 (3.23)	0.008^*^
WHR1	0.91 (0.07)	0.92 (0.04)	0.036^*^
Physical activity (MET·h/per day)^1^	32.67 (12.87)	34.11 (10.92)	0.017^*^
Married, *n* (%)	759 (97.1)	738 (94.4)	0.009^#^
Education level, *n* (%)			
Secondary school or below	416 (53.2)	346 (44.2)	< 0.001^#^
High school or above	366 (46.8)	436 (55.8)	
Household income (yuan/month), *n* (%)			
≤ 2000	273 (34.9)	353 (45.1)	< 0.001^+^
2001–6000	416 (53.2)	349 (44.6)	
> 6000	93 (11.9)	80 (10.2)	
Smoking, *n* (%)	411 (52.6)	362 (46.3)	0.013^#^
Alcohol user, *n* (%)	256 (32.7)	166 (21.2)	< 0.001^#^
Tea Drinker, *n* (%)	428 (54.7)	462 (59.1)	0.083^#^
Energy intake (kcal/d)^2,3^	1513 (1224,1857)	1604 (1301,1950)	< 0.001^+^
Multivitamin use, *n* (%)	44 (5.6)	61 (7.8)	0.086^#^
Hypertension, *n* (%)	111 (14.2)	92 (11.8)	0.153^#^
Diabetes, *n* (%)	67 (8.6)	38 (4.9)	0.003^#^
HBV infection, *n* (%)	678 (87.6)	Not assessed	

^1^Continuous values are mean (standard deviation). ^2^Energy intakes exclude cooking oil. ^3^Values are median (P25, P75). ^*^P values, two-sided *t*-test. ^#^*P* values, two-sided chi-square test. ^+^*P* values, Wilcoxon rank-sum test. Abbreviations: MET = metabolic equivalent. WHR = waist-to-hip ratio.

After the principal components factor analysis (PCFA), we identified three main dietary patterns. The factor loadings associated with each pattern are given in Table 2. We named each pattern according to the food groups with high absolute values of the loadings. The first was labeled the urban prudent dietary pattern (UPDP). This pattern was characterized by high intakes of dairy products, eggs, mushrooms and algae, nuts and soy and a low intake of refined grains. The second pattern was called as the high meat and preserved food pattern (MPFP). It was characterized by high consumption of red meat, animal organ meat, preserved vegetables, cooking oil and processed meat, but lower dairy products. The third pattern represented a high intake of fruits, fish and other seafood, vegetables, Cantonese soup, and Chinese herb tea. Because Cantonese soup and herb tea are common choices in the routine Cantonese diet, this pattern was named as the traditional Cantonese dietary pattern (TCDP). Negative scores signify a lower intake of the specific food within a specific pattern. Higher factor loading scores (and thus higher quartile) signify a higher intake of that particular food group. These three factors explained 11.8%, 9.0%, and 8.4% of the total dietary variability, respectively, and 29.2% of the total variance in food intake overall.

**Table 2:** Varimax-rotated food group factor loading scores^a^

Food Group^b^	Urban Prudent Dietary Pattern	High Fat and Preserved Food Pattern	Cantonese Healthy Dietary Pattern
Refined Grain	–0.772	–0.480	
Dairy products	0.515	–0.414	
Eggs	0.514		–0.286
Mushroom and algae	0.436	–0.251	
Nuts	0.418		
Soyfoods	0.397		
Poultry	0.253		
Red meat		0.561	–0.404
Animal organ meat		0.532	
Preserved vegetable		0.446	0.260
Cooking oil		0.408	
Processed meat		0.259	
Fruits			0.618
Fish and seafood			0.556
Vegetables			0.437
Cantonese Soup			0.386
Chinese herb tea			0.320

^a^Extraction method is principal component analysis. ^b^Factor-loading scores with absolute values below 0.25 are not shown.

Table 3 shows the ORs and 95% CIs for PLC risk according to the quartiles of each dietary pattern. Univariate unconditional logistic regression analyses presented a dose-dependent inverse correlation between risk of liver cancer and the UPDP (*P*-trend < 0.001) and the TCDP (*P*-trend = 0.007), but a significantly adverse relationship with the MPFP (*P*-trend < 0.001). After adjusting for sex, age, BMI, marital status, education level, income level, smoking, alcohol use, tea drinking, physical activity, multivitamin use, and hypertension and diabetes status, significant associations remained. The ORs (95% CI) of PLC risk for the highest quartile (Q4 *VS* Q1) of dietary pattern scores were 0.25, 95% CI: 0.18–0.35; *P*-trend < 0.001) for the UPDP, and 0.61 (95% CI: 0.46–0.82; *P*-trend = 0.002) for the TCDP, showing significantly decreased risks. However, the highest quartiles (*VS*. lowest) of the MPFP were positively associated with the PLC risk, and the OR was 1.98 (95% CI: 1.46–2.69; *P*-trend < 0.001).

**Table 3:** Odds ratios (ORs) and 95% confidence intervals (CIs) of primary liver cancer for quartiles of dietary patterns

	*n*	Univariate model	Model 1
	(cases/controls)	OR (95% CI)	OR (95% CI)
**Urban Prudent Dietary Pattern**			
Q1	295/195	1	1
Q2	240/196	0.81 (0.62–1.01)	0.78 (0.59–1.03)^+^
Q3	151/196	0.51 (0.39–0.67)^***^	0.43 (0.31–0.58)^***^
Q4	96/195	0.33 (0.24–0.44)^***^	0.25 (0.18–0.35)^***^
*P*-trend		< 0.001	< 0.001
**High Meat and Preserved Food Pattern**			
Q1	141/195	1	1
Q2	136/196	0.96 (0.71–1.36)	0.95 (0.69–1.31)
Q3	205/196	1.14 (1.08–1.94)^**^	1.39 (1.02–1.90)^**^
Q4	300/195	2.13 (1.61–2.82)^***^	1.98 (1.46–2.69)^***^
*P*-trend		< 0.001	< 0.001
**Cantonese Healthy Dietary Pattern**			
Q1	250/195	1	1
Q2	181/196	0.72 (0.55–0.95)^*^	0.74 (0.55–0.98)^*^
Q3	182/196	0.72 (0.55–0.95)^*^	0.73 (0.55–0.98)^*^
Q4	169/195	0.68 (0.51–0.89)^**^	0.61 (0.46–0.82)^***^
*P*-trend		0.007	0.002

*p* < 0.001:^***^p < 0.01:^**^p < 0.05:^*^p < 0.1:^+^; Model 1: adjusted for sex, age, BMI, education level, income level, smoking, drinking, drink tea, physical activity, marital status, multivitamin use, and hypertension and diabetes status.

**Figure 1:**
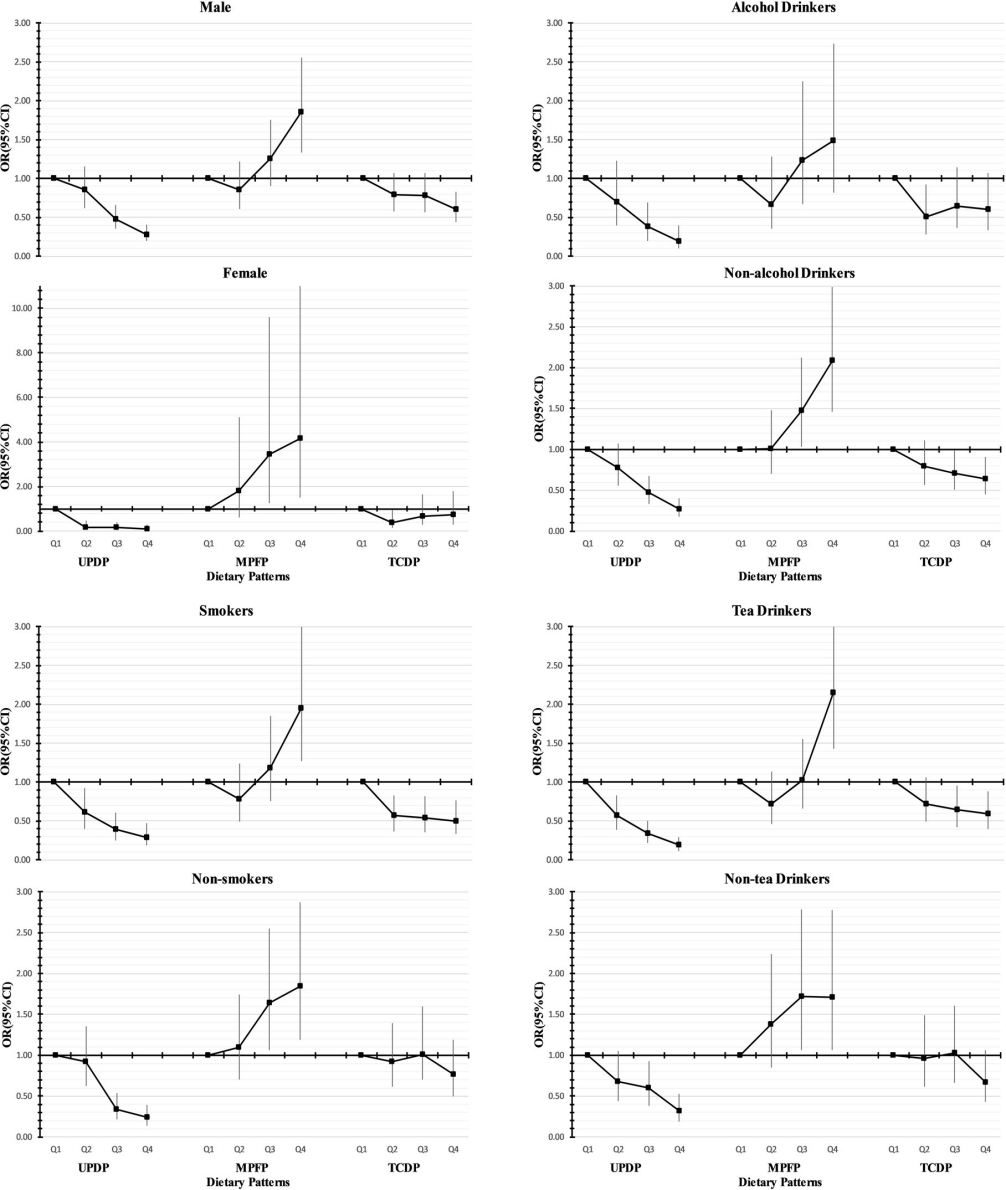
Adjusted Odds ratios (ORs) and 95% confidence intervals (CIs) of primary liver cancer for quartiles of dietary pattern stratified by gender, alcohol use, smoking and tea drinking: adjusted for sex, age, BMI, education level, income level, smoking, drinking, drink tea, physical activity, marital status, multivitamin use, and hypertension and diabetes status

The results of stratified analyses are given in Figure 1. The associations were not significantly modified by gender and the status of smoking, alcohol, or tea drinking (*P*-interaction from 0.118 to 0.914). In some subgroups, such as women, alcohol users, nonsmokers and people who did not drink tea, the favorable effects of the TCDP tended to be insignificant (*P*-trend > 0.05). Because there were no data of HBV infection among controls, we estimated the relevant association according to the HBV infectious status of PLC subjects (yes/no). The adverse association between MPFP and PLC risk tended to be more pronounced in HBV- group than in HBV+ group (Supplementary Table 2). We also conducted sensitive analysis by limited to the HCC subjects and to the subjects without diabetes, similar results were observed in these analyses to those in the total sample (Supplementary Table 3 and Supplementary Table 4).

## DISCUSSION

In this case-control study conducted in China, we identified three major dietary patterns (UPDP, TCDP, and MPFD) in 782 pairs of age- and gender-matched cases and controls via factor analysis and explored their association with the risk of PLC. The first two patterns were associated with lower PLC risk, while the last one correlated to an increased risk of liver cancer.

The first pattern (UPDP), which was associated with a decreased risk of PLC, was characterized by higher consumptions of dairy products, eggs, mushrooms and algae, nuts and soy foods, but lower refined grains. Many studies have focused on the relationship between food groups and cancer risk, but generated inconsistent results. For example, dairy products were correlated to increase risks of hepatocellular carcinoma [10] and endometrial cancer [27], but not to prostate cancer risk [28]. And few studies found an association between nuts, eggs, mushrooms and algae and PLC. However, the characteristics of the urban prudent dietary pattern in our study was analogous to previous studies in which it showed a favorable association with colorectal cancer [29], as well as with other chronic diseases (e.g., cardio-metabolic health [30], hip fractures [31, 32]) and all-cause mortality [30]. Moreover, refined grains was reported to have adverse effect on stomach cancer and colon cancer in previous study [33]. And greater soy consumption was also associated with reduced endometrial cancer risk [34] and stomach cancer death [35]. Considering that difficulty in controlling all potential dietary confounders when conducting an analysis on a single food (or food group), factor analysis may have been an appropriate approach to this issue. One of the main features of this approach is that all dietary constituents added weighted contributions to the factor score, thus making it relatively easy to explain complete variation. This could also explain why we observed a favorable effect of the diet containing the components mentioned above.

In our study, we found that a healthy dietary pattern (TCDP) typified by the consumption of ubiquitous healthy food groups (e.g., fruits, vegetables and fish) and two novel food groups (Cantonese soup and Chinese herb tea) was associated with a 39% decrease (Q4 *VS* Q1) in PLC risk. Inverse association between the consumption of fruit and/or vegetables and hepatocellular carcinoma (HCC) was also observed in many studies, such as the European Prospective Investigation into Cancer and nutrition (EPIC) study [14], the Japan Public Health Center-based Prospective Study (JPHC study) Cohort II [36], the Shanghai Women’s and Men’s Health Studies [37] and the Hiroshima/Nagasaki Life Span Study [38]. Our previous study also showed a favorable association of dietary sources of antioxidants with PLC [13]. Moreover, there is also substantial evidence that foods rich in PUFAs, such as fish, play a role in reducing HCC risk through the anti-inflammatory effect of n-3 PUFAs on chronic hepatitis [17, 18]. The favorable association of this pattern is probably due to the higher intake of antioxidantive and anti-inflammatory phytochemicals and polyunsaturated fatty acids (PUFAs) containing in the foods of this diet pattern. It is well known that cancer progression is closely related to oxidative damage. Liver cancer is also an inflammation-promoted cancer with a background of chronic inflammation induced by either exposure to aflatoxins or chronic hepatitis virus infection. Interestingly, there are also two novel food groups observed in the Cantonese healthy dietary pattern. Studies on the Cantonese diet are very limited, but in Jia *et al*’s study, consumption of herb tea and herbal slow-cooked soup was associated with decreased risk of nasopharyngeal carcinoma, with ORs (Q4 *VS* Q1) of 0.84 (95% CI: 0.68–1.03) and 0.58 (95% CI: 0.47–0.72), respectively [39]. The potential favorable mechanisms of these Cantonese foods requires further research.

Generally consistent with previous studies, we also found that the high meat and preserved food pattern (MPFD) was associated with an increased PLC risk. It is well established that high consumption of red meat, preserved foods and high fat diet increase the risk of cancer. Freedman’s study examined 495,006 men and women from the National Institutes of Health-AARP Diet and Health Study and reported a higher risk of HCC with increased red meat intake (HR _Q5 VS Q1_ = 1.74, 95% CI = 1.16 to 2.61) [8]. Preserved food was reported to increase prostate cancer risk with OR _Q4 VS Q1_ of 7.05 (95% CI = 3.12 to 15.90) [40]. A case-control study conducted in China, the adjusted OR of ovarian cancer is 1.78 (95% CI = 1.35 to 2.34) for the highest relative to the lowest quartile for women intaking more preserved food [41]. Cholesterol was demonstrated to elevate cirrhosis and liver cancer risk (HR _Q4 VS Q1_ = 2.45, 95% CI = 1.34 to 4.7) in the first National Health and Nutrition Examination Survey (NHANES I) [42]. Additionally, the adverse effect of a high-fat diet on liver diseases was even found in a cohort of adolescents with an OR _Q4 VS Q1_ of 1.59 (95% CI = 1.17 to 2.14) [43]. A high fat diet has also been proven to increase liver inflammation and inflammatory cytokines and is related to elevate liver damage and tumor progression in animal models [44].

Some limitations of this study also need to be addressed. First, the restriction of the case-control study design on causality should be considered. The diet-related features of each pattern (e.g., making soup or tea is very time consuming) caused us to consider what other characteristics people would have with high scores in each factor. For instance, did those with high scores in the healthy dietary patterns tend to practice a healthier lifestyle or were they more aware of health care resources? However, these factors neither significantly confounded the association after we adjusted for them in the multivariate models (all *P*-trend < 0.01) nor modified the association (all *P*-interaction > 0.05). Nonetheless, we could not fully control for all the “healthy” lifestyle factors, either through errors in measurement of the exposures or through failure to control for other unknown confounding factors; therefore, residual confounding was still unavoidable as an explanation for the associations. Second, dietary intake information from the FFQ might cause recall bias and contain a certain degree of measurement error, but we attempted to account for the design weaknesses in the following ways. We provided photographs with portion sizes to help participants quantify intake and tried to complete the interview as soon as the diagnosis of the patients was made or before the operation. Actually, the potential bias was unlikely to be differential, because the public was unfamiliar with the studied risk factors for dietary patterns that contain a large variety of different foods, whereas non-differential recall bias tended to attenuate the association to null. Therefore, it is unlikely that we overestimated the favorable association in this study. We also did not include people who had significant dietary and routine activity changes within 5 years before the survey. Third, we could not completely exclude the possibility of reverse causality due to the dietary habit changes caused by the symptoms (e.g., poor appetite and nausea) of liver cancer. The following measures were taken to reduce the short-term influence of PLC on the reported dietary intakes: enrollment of newly diagnosed PLC cases (within 1 month) only, using a FFQ to assess their relatively long-term dietary intake (one year prior to the PLC diagnosis), conducting the face-to-face interview obtaining dietary information by trained and qualified interviewers, and using energy-adjusted food intake to formula dietary patterns. Fourth, we could not adjusted for the HBV status due to lack of the data in the controls. Stratified analyses showed that the adverse association between the MPFP and the PLC risk was much stronger in the HBV- group than in the HBV+ group when using the same controls. Given that the proportion of HBV+ was higher in the PLC subjects than in the controls, the imbalance in the HBV status between PLC case and control groups might underestimate adverse association in this study. In addition, we could not exclude the influence of chronic drug intake due to missing the data. Lastly, we used factor analysis, a data-driven statistical exploratory method. One limitation of this method is that it depends heavily on the study population, which limits the utility of these dietary patterns. However, dietary patterns analogous to our findings were repeatedly reported in a wide range of studies regardless of region, culture, population and FFQ, and the apparent favorable effects mentioned above are also consistent with what we observed. In view of these similarities, it may be reasonable to conclude that our findings reflect reliable dietary patterns found in many populations.

In conclusion, we identified that two “healthy” dietary patterns, the urban prudent dietary pattern and the traditional Cantonese dietary pattern, were favorably associated with the risk of PLC, while the high meat and preserved food pattern was associated with an increased PLC risk in a Chinese population. Our findings provided important clues for the PLC prevention, although further verification is needed in prospective studies.

## MATERIALS AND METHODS

### Study population

All case subjects in this case-control study were recruited in Sun Yat-sen University Cancer Center in Guangzhou (South China) from September 2013 to August 2016. Eligible cases included those patients aged 18 to 80 years; newly diagnosed within one month according to the National Comprehensive Cancer Network (NCCN) Clinical Practice Guidelines in Oncology: Hepatobiliary Cancers [45]; and those who had not undergone any previous treatment. Subjects were excluded for the following: (1) prior history of other cancers; (2) significant changes in dietary habits or routine activities within the previous 5 years; (3) unable to understand Mandarin or Cantonese; and 4) an implausible total daily energy intake (< 700 or > 4200 kcal per day for males, < 500 or > 3500 kcal per day for females). Control subjects in our study were invited from the local communities in Guangzhou through flyers or referrals. Additionally, controls included in our study were age- and sex-matched with cases and required to meet the same inclusion and exclusion criteria (except for liver cancer). Eventually, a total of 782 new cases and 782 controls were included in this study. The Ethics Committee of the School of Public Health at Sun Yat-sen University approved the study protocol. Written informed consent was obtained from all participants at initial enrollment.

### Data collection

A structured questionnaire was used to investigate participants’ dietary behaviors and confounding factors through an in-person interview conducted by trained interviewers. The following information was collected: anthropometric measurements (current height and weight, circumferences at the waist, hip, and neck) socio-demographic characteristics (e.g., age, gender, marital status, education level, occupation, household income); lifestyle habits (e.g., smoking, alcohol use, tea drinking, multivitamin use); medical histories; physical activity level and habitual dietary intake in the year prior to the interview. Education level was grouped into two levels: secondary school or below and high school or above. Household income was grouped into three levels: (*≤* 2000, 2001 – 6000, and > 6000 Yuan/month/person). Individuals who smoked more than one cigarette daily or drank alcohol more than once weekly continuously for at least six months were considered smokers or alcohol users. Tea drinkers were defined as participants who drank tea at least twice a week. Physical activity was reported as metabolic equivalent hours per day (MET h/day), measured by a 19-item questionnaire covering questions about daily, occupational, and leisure time activities. Body mass index (BMI, kg/m^2^) and waist-to-hip ratio (WHR) were also calculated for further analysis.

### Dietary assessment and food group identification

Dietary consumption was evaluated by an interviewer-administered 79-item food frequency questionnaire (FFQ) that has been validated in previous studies [13]. All participants were required to report their intake frequency (never, per year, per month, per week, or per day) and the average amount of each food item. Photographs of common foods in usual portion sizes were offered to participants to assist with quantifying intake. Each item was converted to the daily intake of grams per day, and the average daily intake of nutrients and total energy was calculated according to the Chinese Food Composition Table 2004 [46]. The 79 food items were first aggregated into 17 food groups to increase the clarity of the information (Supplementary Table 1). Results were aggregated according to the similarity of the nutrient profiles, and factor analysis was then used to derive the dietary patterns, which could generate a small number of variables that reflected the information from the larger data sets.

### Statistical analysis

We used the *t*-test, chi-squared test, and Wilcoxon rank-sum test to test differences in socio-demographic and other potential PLC risk factors between cases and controls as appropriate. Intake of each food item was adjusted for total energy intake in a combination of cases and controls using the residual method [47]. Three factors that capture the primary sources of variation in the reported FFQ were retained based on the inspection of scree plots via principal components factor analysis (PCFA). The factors were rotated using the varimax procedure (orthogonal transformation) to facilitate their interpretability. For every subject, we calculated factor scores on each of the 3 retained factors by summing the intakes of each food group multiplied by their factor loadings. Quartiles of the factor scores were defined based on the distribution among controls and were used for comparison with nutrient intake to estimate their associations with PLC. Logistic regression was applied to estimate odds ratios (ORs) and the corresponding 95% confidence intervals (CI). The bottom quartile group (Q1) was defined as the reference. We used both univariate and multivariate analyses. In multivariate analyses, we adjusted for sex, age, body mass index (BMI), physical activity (MET h/day), marital status (yes or no), education level (secondary school or below and high school or above), income level (≤ 2000, 2001–6000, > 6000 Yuan/month/person), currently smoking (yes or no), currently using alcohol (yes or no), currently drinking tea (yes or no), multivitamin use, and hypertension and diabetes status. To test linear trends, quartiles for each factor group were treated as continuous variables in the regression analyses.

We also conducted stratified analyses to test whether the above-mentioned associations were modified by gender (male *VS* female) and lifestyle habits (smoking, alcohol use, and tea drinking). Multiplicative interactions were estimated by adding interaction terms according to the likelihood ratio test. All data were analyzed using IBM SPSS Statistics version 20.0. (Armonk, NY: IBM Corp., USA). *P* values were based on two-sided tests and were considered significant at < 0.05.

## SUPPLEMENTARY MATERIALS TABLES



## Abbreviations

PLC: Primary liver cancer
UPDP: Urban prudent dietary pattern
TCDP: Traditional Cantonese dietary pattern
MPFP: High meat and preserved food pattern
HBV: Hepatitis B virus
HCV: Hepatitis C virus
BMI: Body mass index
WHR: Waist-to-hip ratio
PCFA: Principal components factor analysis
ORs: Odds ratios
CI: Confidence Interval
PUFAs: Polyunsaturated fatty acids
HCC: Hepatocellular carcinoma
EPIC: European Prospective Investigation into Cancer and nutrition study
JPHC: Japan public health center-based prospective study
NHANES: National Health and Nutrition Examination Survey
NCCN: National Comprehensive Cancer Network
MET: Metabolic equivalent
FFQ: Food frequency questionnaire
