# Supplementary material for: Dietary patterns and primary liver cancer in Chinese adults: a case-control study
Source: Oncotarget. 2018 Jan 4;9(45):27872–81. doi: 10.18632/oncotarget.23910 (PMC6021235; doi:10.18632/oncotarget.23910)
Supplement: Supplementary file 1 [file oncotarget-09-27872-s001.pdf]

Dietary patterns and primary liver cancer in Chinese adults: a case-control study

SUPPLEMENTARY MATERIALS

Supplementary Table 1: Food groups included in the factor analysis

Food groups	Items
Refined Grain	White rice, porridge, noodles, bread, cake, biscuits
Vegetables	Dark green leafy vegetables, cruciferous, vegetables, melon, radish and pepper, carrot, tomato, starchy tubers, fresh corn, fresh beans, allium
Soyfoods	Hard tofu, soft tofu, fried tofu pop, tofu curd, vegetarian chicken, bean curd pudding, soy milk, fresh soybean, and dried soybean
Fruits	Citrus fruits, apple, pear, peach, plum, banana, grape, litchi, longan, watermelon, papaya, cantaloupe, kiwi fruit, strawberry, pineapple, mango, durian
Red meat	Pork, beef, lamb
Animal organ meat	Liver, kidney, brain
Processed meat	Sausage, ham, bacon
Preserved vegetable	Salt mustard greens, preserved Szechuan pickle
Poultry	Chicken with or without skin, duck, goose
Fish and seafood	Fresh water fish, salt water fish, canned fish, shrimp, crab, squid, cuttle, scallops, mussel, whelk
Eggs	Egg, duck egg
Dairy products	Whole milk, whole milk powder, skim/ low-fat milk, skim/ low-fat milk powder, yoghurt, cheese, milk tea, ice cream
Nuts	Peanut, cashew nut, walnut, gingko, almond, pistachio nuts and sesame
Mushroom and algae	Mushroom and algae
Cooking oil	Cooking oil
Cantonese soup	Chinese double-stewed soup
Chinese herb tea	Chinese herb tea

Supplementary Table 2: Associations between quartiles of dietary patterns by controls and PLC risk according to HBV infectious

		Q1	Q2	Q3	Q4	*P*-trend
**Urban Prudent Dietary Pattern**						
HBV infectious						
Yes	*n* (cases/controls)	261/195	207/196	133/196	77/195	
	OR (95% CI)[1]	1	0.76 (0.57–1.01)$^{+}$	0.44 (0.32–0.60)***	0.24 (0.16–0.34)***	< 0.001
No	*n* (cases/controls)	34/195	33/196	18/196	19/195	
	OR (95% CI)[1]	1	0.84 (0.48–1.48)	0.37 (0.19–0.73)**	0.35 (0.18–0.69)**	< 0.001
**High Meat and Preserved Food Pattern**						
HBV infectious						
Yes	*n* (cases/controls)	130/195	116/196	176/196	256/195	
	OR (95% CI)[1]	1	0.86 (0.62–1.20)	1.24 (0.90–1.71)	1.77 (1.29–2.42)***	< 0.001
No	*n* (cases/controls)	11/195	20/196	29/196	44/195	
	OR (95% CI)[1]	1	2.30 (1.02–5.20)*	3.73 (1.69–8.24)**	5.36 (2.45–11.73)***	< 0.001
**Cantonese Healthy Dietary Pattern**						
HBV infectious						
Yes	*n* (cases/controls)	223/195	158/196	158/196	139/195	
	OR (95% CI)[1]	1	0.73 (0.55–0.99)*	0.72 (0.54–0.97)*	0.58 (0.43–0.79)***	0.001
No	*n* (cases/controls)	27/195	23/196	24/196	30/195	
	OR (95% CI)[1]	1	0.79 (0.43–1.49)	0.78 (0.42–1.47)	0.85 (0.46–1.58)	0.642

$p < 0.001$:***; $p < 0.01$:**; $p < 0.05$:*; $p < 0.1$:$^{+}$

[1]: adjusted for sex, age, BMI, education level, income level, smoking, alcohol use, tea drinking, physical activity, marital status, multivitamin use, and hypertension and diabetes status.

Supplementary Table 3: Associations between quartiles of dietary patterns by controls and PLC risk in hepatocellular carcinoma patients

	Q1	Q2	Q3	Q4	*P*-trend
**Urban Prudent Dietary Pattern**					
n (cases/controls)	242/195	202/196	121/196	72/195	
OR (95% CI)[1]	1	0.81 (0.61–1.08)	0.43 (0.31–0.59)***	0.24 (0.16–0.34)***	< 0.001
**High Meat and Preserved Food Pattern**					
n (cases/controls)	117/195	115/196	155/196	250/195	
OR (95% CI)[1]	1	0.96 (0.69–1.35)	1.27 (0.91–1.76)	2.00 (1.45–2.76)***	< 0.001
**Cantonese Healthy Dietary Pattern**					
n (cases/controls)	204/195	157/196	156/196	120/195	
OR (95% CI)[1]	1	0.80 (0.59–1.08)	0.78 (0.58–1.06)	0.55 (0.40–0.76)***	< 0.001

$p < 0.001$:***; $p < 0.01$:**; $p < 0.05$:*; $p < 0.1$:$^{+}$

[1]: adjusted for sex, age, BMI, education level, income level, smoking, alcohol use, tea drinking, physical activity, marital status, multivitamin use, and hypertension and diabetes status.

Supplementary Table 4: Associations between quartiles of dietary patterns by controls and PLC risk in subjects without diabetes

	Q1	Q2	Q3	Q4	*P*-trend
**Urban Prudent Dietary Pattern**					
n (cases/controls)	268/186	223/186	133/186	91/186	
OR (95% CI)[1]	1	0.80 (0.60–1.07)	0.43 (0.31–0.59)***	0.27 (0.19–0.38)***	< 0.001
**High Meat and Preserved Food Pattern**					
n (cases/controls)	139/186	119/186	187/186	270/186	
OR (95% CI)[1]	1	0.83 (0.59–1.15)	1.30 (0.94–1.78)	1.80 (1.32–2.46)***	< 0.001
**Cantonese Healthy Dietary Pattern**					
n (cases/controls)	226/186	168/186	174/186	147/186	
OR (95% CI)[1]	1	0.75 (0.56–1.01)$^{+}$	0.77 (0.57–1.03)$^{+}$	0.57 (0.42–0.77)***	0.001

$p < 0.001$:***; $p < 0.01$:**; $p < 0.05$:*; $p < 0.1$:$^{+}$

[1]: adjusted for sex, age, BMI, education level, income level, smoking, alcohol use, tea drinking, physical activity, marital status, multivitamin use, and hypertension and diabetes status.
